# Reassessment of two finite-element studies of infant head trauma: limits of the modelling evidence for shaking-induced bridging-vein rupture and protection by enlarged subarachnoid spaces

**DOI:** 10.3389/fbioe.2026.1853681

**Published:** 2026-08-04

**Authors:** Cyrille Rossant, Keith D. Button

**Affiliations:** 1 Queen Square Institute of Neurology, University College London, London, United Kingdom; 2 Explico Inc., Novi, MI, United States

**Keywords:** benign enlargement of the subarachnoid spaces, bridging veins, finite-element analysis, infant head injury biomechanics, model validation, subdural hemorrhage, verification and validation

## Abstract

**Introduction:**

Finite-element (FE) models are often invoked to study infant head trauma where direct experimentation is impossible, but their evidentiary value depends on credible geometry, loading, constitutive assumptions, verification, and validation. We present a biomechanical reassessment of two influential FE studies published in 2007 and 2008 that concluded, respectively, that shaking without impact can produce bridging-vein loading comparable to impact and that benign enlargement of the subarachnoid space (BESS) protects against subdural hemorrhage.

**Methods:**

Treating these papers as modelling evidence, we re-examined their reported geometry, loading, vein representation, and inferential logic in light of later work on infant head kinematics, bridging-vein morphology and mechanics, and pediatric FE credibility.

**Results:**

Neither paper reported mesh or parameter sensitivity, validated brain motion or venous response, or demonstrated that the implemented loads reproduced the head kinematics of the surrogate experiments used to justify them. The shaking input was inherited from a worst-case surrogate with a resistance-free sagittal hinge neck, whereas the comparison impact was a lower-severity, non-rotational loading condition. Bridging veins were represented as straight linear springs, and the BESS analysis depended on a zero pre-strain assumption that is not biologically valid.

**Discussion:**

These limitations mean that the two studies do not provide robust modelling support for the claims that shaking alone produces impactequivalent bridging-vein loading or that enlarged subarachnoid spaces protect against subdural hemorrhage. We conclude by identifying minimum credibility requirements for pediatric FE studies used to support forensic or clinical inference.

## 1 Introduction

Pediatric head injury is a central concern in clinical care and forensic practice because of its potential for severe neurological sequelae and legal consequences. The contemporary discourse on the particularly severe form of non-accidental trauma termed shaken baby syndrome/abusive head trauma (SBS/AHT) emerged from child-protection efforts and early medical descriptions of injury patterns in young children (Kempe et al., 1962; Guthkelch, 1971; Caffey, 1972). Specifically, early work often treated the combination of subdural hematomas (SDH), retinal hemorrhages (RH), and encephalopathy in infants as indicative of a violent shaking event (Sirotnak and Krugman, 1994; Kirschner, 1997).

Because ethical constraints preclude controlled human experimentation, the field has necessarily relied on biomechanics, physical surrogates, and computational modelling to investigate injury mechanisms (Gennarelli, 1985; Duhaime et al., 1987; Ommaya et al., 2002; Goldsmith and Plunkett, 2004; van Zandwijk et al., 2019; Vester et al., 2019). In that setting, the question is not whether FE models are useful in principle, but whether a given model is sufficiently credible for the mechanistic inference drawn from it.

In France, national guidance issued by the *Haute Autorité de Santé* (HAS) has structured diagnostic pathways for suspected shaking since 2011 (HAS, 2011; 2017). These guidelines have given unusual practical weight to two FE studies published in 2007 and 2008, which have been cited in both medical and legal settings (Rossant and Schneps, 2020). Because a new revision of the guidance is underway (Rossant and Etrillard, 2023), reassessing the modelling basis of these papers is timely.

The first study compared a simulated shaking event with a simulated inflicted impact and reported that both scenarios stretched infant bridging veins (BV) by comparable amounts (Roth et al., 2007). Relying on the assumption that inflicted impact is expected to rupture BV and thereby cause subdural bleeding, the authors inferred that shaking without impact could produce the same injury outcome.

The second paper modified the same head model to represent benign enlargement of the subarachnoid space (BESS), a condition characterized by enlargement of the cerebrospinal-fluid-filled space between the brain and the dura. Although many clinical studies over the past half century have consistently noted an increased risk of SDH in infants with BESS—presumably due to increased stretch and fragility of BV traversing the widened pericerebral spaces—this numerical study reported that a wider CSF layer reduces relative brain–skull motion (Raul et al., 2008). This finding has been cited as the principal justification within the HAS guidelines for dismissing BESS as a risk factor for SDH in infants.

The present paper revisits the modelling choices, loading conditions, and interpretations presented in these two FE studies nearly 2 decades later, after substantial developments in pediatric head biomechanics, model credibility standards, and the clinical literature on enlarged subarachnoid spaces. Our aim is not to resolve the entire clinical controversy around infant head injury, but to assess whether these particular numerical studies provide robust support for the conclusions attributed to them. We therefore focus on model construction and verification, the realism of the imposed shaking and impact scenarios, the treatment of bridging veins and their inferred failure, and the assumptions surrounding the BESS simulations.

We evaluate modelling credibility rather than epidemiology: geometry and material choices, loading realism, bridging-vein representation, and the presence of verification/validation (VandV) and uncertainty analyses. Contemporary credibility expectations include sensitivity analyses for mesh, contact, and material choices, together with validation of key responses against experimental corridors. For pediatric heads, global impact validation has been demonstrated for skull response (Roth et al., 2010), while realistic shaking inputs should reflect measured head–neck kinematics with moving centres of rotation and contact events where relevant (Reimann, 2018; Kleiven et al., 2022; Schiks et al., 2023; Miyazaki, 2024). The issue for the present paper is therefore one of computational biomechanics: whether the numerical formulation, loading implementation, and validation evidence are adequate for the specific claims made about venous loading and BESS.

Finally, because the 2008 BESS conclusion has influenced French guidance, we note early that clinical cohort and imaging data generally report elevated prevalence of subdural collections in infants with enlarged subarachnoid spaces, contrary to a protective interpretation (Tucker et al., 2016; Caré, 2021; Alshareef et al., 2022; Baig et al., 2022; Scheller and Wester, 2022). This motivates a careful re-examination of the BESS modelling assumptions in the reassessed articles.

## 2 Materials and methods

We reviewed the original publications by (Roth et al., 2007; Raul et al., 2008), focusing on modelling credibility rather than on resolving the clinical diagnosis of abusive head trauma. While neither paper reported mesh or parameter sensitivity, we extracted the reported geometry, constitutive assumptions, boundary conditions, loading definitions, and inferential steps linking model output to claims about bridging-vein rupture and BESS. Where possible, we checked internal consistency by converting reported input velocities to equivalent fall severities, by recomputing the published vein-stretch relations under alternative initial conditions, and by comparing the imposed shaking and impact inputs with the surrogate experiments and later kinematic literature used to justify them.

To benchmark the 2007–2008 FE assumptions, we conducted a targeted literature review focused on three domains: infant shaking and impact kinematics, bridging-vein morphology and mechanics, and verification/validation (VandV) expectations for pediatric head FE models. Sources were identified from prior reviews (van Zandwijk et al., 2019; Vester et al., 2019) and from experimental and computational studies directly relevant to the assumptions embedded in the original papers (Duhaime et al., 1987; Cory and Jones, 2003; Prange et al., 2003; Roth et al., 2010; Lloyd et al., 2011; Jones et al., 2015; Jenny et al., 2017).

### 2.1 Literature search and selection

We used a keyword-based approach to identify relevant biomechanics and FE studies from review articles and their reference lists. We prioritized infant head kinematics in falls and impacts (Bertocci et al., 2003; 2022; Coats and Margulies, 2008; Ibrahim and Margulies, 2010), bridging-vein mechanics and morphology (Löwenhielm, 1974; Lee and Haut, 1989; Monson et al., 2003; Depreitere et al., 2006; Mortazavi et al., 2013; Famaey et al., 2015), and FE VandV expectations (Roth et al., 2010; Jones et al., 2017). We also considered studies that bound shaking severity or reviewed the limitations of existing models (Reimann, 2018; van Zandwijk et al., 2019; Vester et al., 2019; Kleiven et al., 2022; Schiks et al., 2023). Selection was non-systematic but aimed to capture variability directly relevant to the original papers’ assumptions and inferences.

### 2.2 Analysis approach

We compared the original modelling choices and inputs informally with ranges reported in the literature. Since the shaking and impact inputs for the (Roth et al., 2007) study were based on the results of a 2003 experimental study by (Prange et al., 2003), our kinematic considerations included not only published infant ATD tests and video-derived falls but also the stated design intent of the Prange surrogate. Specifically, Prange et al. explicitly used a resistance-free sagittal hinge neck to approximate a worst-case upper bound (Bertocci et al., 2003; 2022; Prange et al., 2003; Coats and Margulies, 2008). Regarding the modelling of bridging veins, we contrasted the linear spring representation with descriptions of venous geometry, orientation, sinus cuff anatomy, and mechanical behaviour (Löwenhielm, 1974; Monson et al., 2003; Depreitere et al., 2006; Mortazavi et al., 2013; Famaey et al., 2015) For credibility, we distinguished three levels of support: numerical verification, validation of internal responses, and demonstration that the imposed loads reproduce the external kinematics invoked to justify them. Simple equivalences such as *v* ^*2*^ *= 2gh* were used only to contextualize the reported inputs, not as substitute injury criteria.

## 3 Results

### 3.1 Model credibility and validation

Both papers state that the infant head model was created from a single six-month-old CT scan, with no comparison against experimental measurements of skull deformation, brain motion, or venous response (Roth et al., 2007; Raul et al., 2008). Neither article reported mesh-independence checks, contact tuning, time-step checks, or parameter sensitivity studies. Material properties for skull, sutures, membranes, and brain were taken from adult or animal sources without uncertainty propagation. Bridging veins were implemented as straight linear springs whose rest length equals the cerebrospinal fluid (CSF) gap. Subsequent work has demonstrated that replacing linear elastic models for suture, scalp, and dura with experimentally derived nonlinear constitutive models was necessary to reproduce cadaveric drop test responses across multiple impact locations, with any single linear elastic tissue substitution producing acceleration errors of 20%–50% (Li et al., 2017). Additionally, subsequent work has proven the efficacy of subject-specific and anatomically detailed models for both adults (Li et al., 2021) and infants (Li et al., 2019). Recent work has also presented validation data for brain motion and strain (Alshareef et al., 2018; Zhou et al., 2019). The central injury claim in the 2007 paper and the central protective claim in the 2008 paper therefore both relied on output from a model that was neither numerically verified in the usual FE sense nor validated for the local mechanism being interpreted.

### 3.2 Loading realism and kinematic fidelity

The shaking simulation applied one angular-velocity cycle drawn from (Prange et al., 2003) and assumed a fixed rotation centre at the C5–C6 level (Roth et al., 2007). This choice imported a specific surrogate design whose neck was intentionally built as a resistance-free sagittal hinge to produce a worst-case upper bound on head rotation rather than a validated representation of infant neck mechanics (Prange et al., 2003). The Prange authors explicitly noted that this simplification overestimates rotational motion and excludes translation, out-of-plane motion, damping, and moving centres of rotation. Using that signal as a direct FE input therefore embeds an upper-bound surrogate assumption into the head model before any internal response is computed.

The 2007 paper also does not show that its FE implementation reproduces the global head kinematics of the surrogate study on which it relies. The Prange experiments reported shaking angular accelerations on the order of 2640 rad/s^2^ and substantially higher peaks for inflicted impacts against hard surfaces (on the order of 89,400 rad/s^2^) (Prange et al., 2003). Yet the FE paper does not demonstrate that its imposed one-cycle shaking waveform and its prescribed impact loading generate corresponding head-level kinematics in the model. This omission matters because the inferential chain depends not just on the source experiment, but on faithful transfer of that experiment into the FE domain.

**FIGURE 1:**
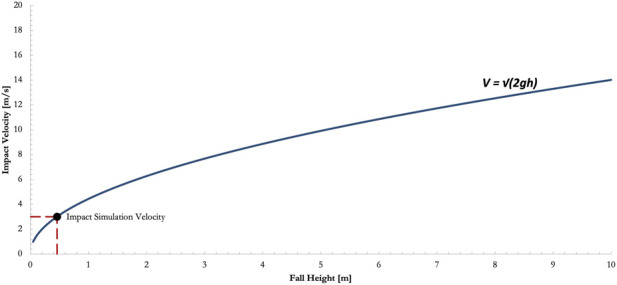
Chart depicting impact velocity versus fall height and the selected impact velocity (3 m/s) for the 2007 impact simulations.

In contrast to the shaking case, the impact simulation prescribed only a horizontal velocity of 3 m/s against a rigid wall, with no rotational component. A 3 m/s translation corresponds to the impact speed from a free fall of height *h* given by *v* ^*2*^ *= 2gh*, i.e., *h = v* ^*2*^ */(2g) ≈ 9/(2 × 9.81) m ≈ 0.46* m (Figure 1). The cited surrogate experiments, however, defined inflicted impact as the terminal contact portion of a vigorous shake and reported combined translational and rotational motions, with the hardest impacts exceeding even a 1.5 m fall onto concrete in angular response (Prange et al., 2003). The FE impact is thus better understood as a materially different, lower-severity loading condition than the surrogate impact used to motivate the comparison, rather than as a low-speed version of the same event.

Given these unbalanced inputs, claiming comparable BV strain magnitudes for shaking and impact effectively equates the chosen shaking case to a non-rotational impact at about the speed of a 0.46 m free fall. The paper therefore compared an upper-bound shaking surrogate input with a reduced impact surrogate rather than two matched mechanisms. That comparison may yield similar strains in this model, but it cannot support a general claim that shaking and inflicted impact are biomechanically equivalent with respect to bridging-vein loading. It also sits uneasily with French guidance that discounts short falls as explanations for SDH in infants (HAS, 2011).

Furthermore, recent experiments and modelling show that the infant head’s instantaneous center of rotation (ICOR) moves substantially during shaking (Schiks et al., 2023). Fixed-ICOR assumptions, employed in the Prange experiments, simplify implementation but can bias skull–brain relative motion and predicted BV stretch. In the present context, the issue is broader than ICOR alone: the imported Prange waveform derives from an upper-bound hinge-neck surrogate that suppresses translation and out-of-plane motion, while the FE studies did not show that the resulting head kinematics in their own model remained faithful to the source experiment. Internal strain fields inferred under such conditions cannot be treated as robust representations of real infant shaking.

### 3.3 Morphological context for bridging veins

Contemporary reviews describe BV complexes as heterogeneous structures with variable orientation, slack, and a specialized cuff near the superior sagittal sinus, exhibiting nonlinear and viscoelastic behavior (Mortazavi et al., 2013; Famaey et al., 2015). Autopsy and histological examinations further document numerous, small-diameter bridging veins, marked inter-individual variability, and peri-sinus cuff features (Cheshire et al., 2018; 2024). In a survey of 43 paediatric post-mortems, the mean number of bridging veins mapped per brain was 54.1 (maximum 94) and measured vein diameters ranged from 0.05 mm to 3.07 mm with a mean of 0.93 mm (Cheshire et al., 2018). Linear spring elements cannot capture geometry-dependent slack, directional loading, rate effects, or cuff stress concentrations across such a heterogeneous network. Consequently, the absolute strain values reported on such springs should not be interpreted as direct evidence of BV rupture risk without a more realistic constitutive and geometric formulation.

### 3.4 Bridging-vein representation and failure inference

The springs representing bridging veins reached 90%–100% engineering strain in the shaking run and about 100% during the impact (Roth et al., 2007). Interpreting these values as rupture evidence requires three assumptions at once: that the vein geometry is adequately represented, that the material law is adequate, and that the chosen strain metric tracks failure. None is secure here.

**FIGURE 2:**
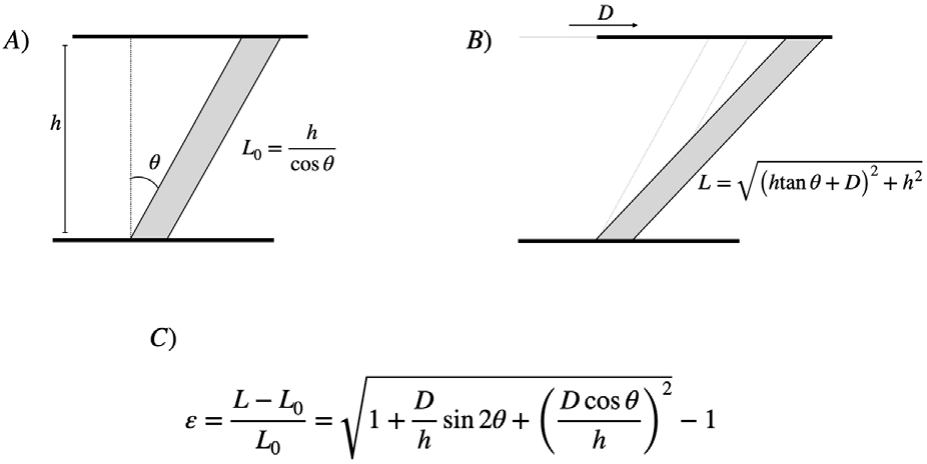
Modelling of a bridging vein **(A)** with the skull subjected to a displacement **(B)**, and the resulting expression for strain **(C)**.

**FIGURE 3:**
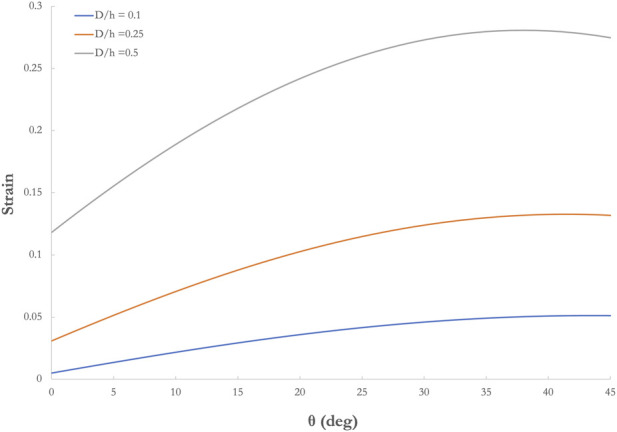
Strain versus bridging vein angle (θ) for D/h ratios of 0.1, 0.25, and 0.5.

Geometrically, linear springs cannot capture the free bridging segment, cuff region near the superior sagittal sinus, or directional dependence of venous loading (Depreitere et al., 2006; Mortazavi et al., 2013; Famaey et al., 2015). Depreitere and colleagues noted that many veins over the posterior frontal, parietal, and occipital convexity run anteriorly toward the superior sagittal sinus. That orientation makes occipital loading especially effective at generating longitudinal strain when the brain lags behind the skull, which is precisely the kind of directional effect erased by straight springs lacking anatomical course (Depreitere et al., 2006). This phenomenon is illustrated in Figures 2, 3, demonstrating how when a vein of length (L_0_), oriented at an angle (θ), is subjected to a skull displacement (D), the resulting strain will increase with increasing θ (for θ < ∼45°). This directional-dependence is important and was not modelled in Roth and Raul’s studies.

Materially, experimental and review data describe nonlinear, viscoelastic, and rate-sensitive behaviour rather than a single linear response (Löwenhielm, 1974; Monson et al., 2003; Famaey et al., 2015). Because the simulated impact involves faster loading than the shaking cycle, a rate-insensitive spring response is not a reliable proxy for venous mechanics. spring strain therefore remains only a surrogate measure here, and it has not been validated as a failure metric for infant bridging veins under cyclic, low-frequency loading.

### 3.5 CSF modelling

A further unaddressed limitation in both studies concerns the representation of CSF in the subarachnoid space. In both (Roth et al., 2007; Raul et al., 2008), the CSF is modelled as a single solid layer with a Young’s modulus of 12 kPa and a Poisson’s ratio of 0.49. This implementation is problematic on multiple grounds. First, the assigned stiffness of 12 kPa exceeds the shear stiffness typically in infant head models, meaning the layer separating the brain from the skull is artificially stiffer than the tissue it surrounds. Second, a Poisson’s ratio of 0.49 in a standard finite element formulation produces a bulk-to-shear modulus ratio approaching incompressibility, which would not be accurate for the modelling application. Third, and most consequentially for the injury mechanism being modelled, merging solid CSF elements to the brain surface eliminates tangential sliding at the skull–CSF–brain interface entirely. Because relative motion between the brain cortex and the overlying dura is the primary kinematic driver of bridging vein stretch, suppressing it by design constrains the very output the models are used to interpret.

Zhou et al. (2020) directly evaluated the consequences of this class of modelling choice by comparing four brain–skull interface approaches—solid elements merged or sliding, and fluid elements merged or sliding—in their response to an applied loading known experimentally to cause SDH. The authors concluded that the CSF needed to be modelled as a fluid with sliding elements against the brain in order to reproduce the clinically observed pattern of SDH risk. Thus, by locking the brain to the skull through a solid, overly stiff CSF layer with no permitted sliding, both Roth and Raul models structurally suppressed the relative motion that would stretch the parasagittal bridging veins. Conclusions about the sufficiency or insufficiency of a given loading to rupture bridging veins cannot be accurately drawn from models in which the brain–skull interface is represented in this way.

### 3.6 BESS assumptions and CSF widening

**FIGURE 4:**
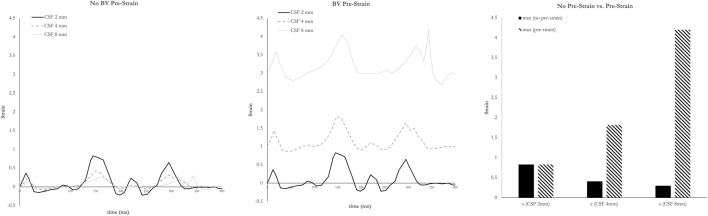
Comparison of the calculated strains from Raul, 2008 with corrected strains accounting for a pre-strain assumption.

The BESS simulations enlarged the CSF layer uniformly from a 2 mm baseline, described by the authors as corresponding to the 50th percentile, to 4 and 8 mm while keeping the brain volume and all material properties fixed (Raul et al., 2008), but clinical imaging shows non-uniform widening around the convexities rather than a uniform shell (Caré, 2021; Scheller and Wester, 2022). The 8 mm width corresponds to a head circumference of about 55 cm, which is unrealistic. For context, the 97th percentile head circumference for a 5-year old is 53 cm (https://cdn.who.int/media/docs/default-source/child-growth/child-growth-standards/indicators/head-circumference-for-age/cht_hcfa_boys_p_0_5.pdf?sfvrsn=1761a85f_7). The authors reported smaller peak strains in the bridging-vein springs and described this as a damping effect. However, the springs were assumed to have zero initial strain regardless of CSF width. If a vein of undeformed length *l*_*0*_ is gently stretched to match the wider gap before shaking begins, the strain during shaking becomes

$$\varepsilon =\left(l-l_{0}\right)/l_{0}$$

where *l* is the peak length predicted during motion. The calculation applies the engineering strain definition to the initial, widened gap: for each scenario, the vein’s undeformed length *l*_*0*_ is taken as the initial CSF gap used in the model (e.g., 2 mm, 4 mm, or 8 mm), and *l* is the peak length reported for the spring during motion. If the *l*_*0*_ is set to 2 mm instead of the CSF gap (i.e., accounting for any pre-strain existing within the spring), the corresponding engineering strains are approximately 0.9, 1.8, and 3.9 for the 2 mm, 4 mm, and 8 mm cases, respectively. In other words, a reasonable amount of pre-strain reverses the trend reported in the paper and suggests that wider CSF spaces could increase, rather than reduce, the stretch on bridging veins (Figure 4).

The rationale given for setting zero initial strain was that BESS develops gradually, so any elongation would occur quasi-statically and without appreciable tension at the start of a simulated shaking event (Raul et al., 2008). This effectively treated each bridging vein’s rest length as equal to the expanded subarachnoid gap. That assumption is questionable on both anatomical and mechanical grounds. Bridging veins show a variable course with a free bridging segment, cuffs and tethering near the sinus, and nonlinear load response (Mortazavi et al., 2013; Famaey et al., 2015). Slow geometric enlargement does not imply zero pre-stress, because venous walls and attachments can still carry baseline tension needed to maintain patency and geometry (Papasian and Frim, 2000). Imaging and morphometric reports also indicate vein displacement in craniocerebral disproportion, consistent with nonzero pre-strain before superimposed motion (Caré, 2021; Scheller and Wester, 2022). A stronger analysis would therefore have tested plausible ranges of initial strain and slack. Under such alternatives, the reported BESS “damping” effect can reverse, as the simple calculation above illustrates.

### 3.7 Clinical context for BESS

Clinical series and cohorts report an association between benign enlargement of the subarachnoid spaces (BESS) and subdural collections or haemorrhage in infancy. In a large imaging cohort for macrocephaly (Tucker et al., 2016), found incidental subdural collections in *∼*6% of infants with BESS, with higher BESS grades associated with increased prevalence and an adjusted odds ratio of 3.68. Population and single-centre studies describe subdural collections in BESS or macrocrania cohorts (Kapila et al., 1982; Greiner et al., 2013; Caré, 2021; Alshareef et al., 2022). Case series note that collections may follow minor or unrecognized trauma (Piatt, 1999; Vinchon et al., 2010; Zahl et al., 2011).

Recent reviews emphasize diagnostic pitfalls: BESS can coexist with subdural collections and mimic abusive head trauma unless macrocephaly trajectories and imaging signs (e.g., arachnoid plane, vein displacement) are recognized (Caré, 2021; Baig et al., 2022; Scheller and Wester, 2022). Taken together, the clinical evidence contradicts the conclusion in (Raul et al., 2008) that wider CSF spaces reduce bridging-vein strain and risk. Instead, clinical data more consistently indicate increased susceptibility to subdural collections in BESS, in line with geometric reasoning and pre-strain effects (Papasian and Frim, 2000). While we do not attempt to resolve the entire clinical controversy, this contradiction between the Raul findings and real-world observations underscores the need to reassess the model findings.

### 3.8 Kinematic benchmarks and documented events

Physical studies of infant surrogates provide context for shaking inputs. Replications and variants of the Duhaime protocol (Duhaime et al., 1987) using different necks and ATD designs found that non-contact shaking produces lower linear and angular accelerations than those reported for impacts (Glowinski et al., 2021; Lee-Confer et al., 2025), and sometimes comparable to vigorous activities of daily living (ADLs (Cory and Jones, 2003; Lloyd et al., 2011). Peaks from shaking increase when chin–chest or head–torso contact occurs (Jenny et al., 2017), but such contacts depend strongly on surrogate mass, stiffness, and setup and may be biologically implausible to sustain repeatedly in real infants (Johnson and Auer, 2018). Across fall studies, peak kinematics are dominated by impact contact (Bertocci et al., 2003; Coats and Margulies, 2008), underscoring the importance of representing contact events when comparing shaking and impact scenarios.

Documented impact events, including witnessed and video-recorded accidental short (<1 m) falls show that SDH and severe retinal haemorrhages can occur in real-world impact scenarios (Aoki, 1984; Plunkett, 2001; Gardner, 2007; Lantz and Couture, 2011; Shuman and Hutchins, 2017; Atkinson et al., 2018; Geoghegan et al., 2023; Brook et al., 2024). These contextual observations are secondary, and the main issue remains the credibility of the FE studies themselves.

## 4 Discussion

The central point of this reassessment is not that the 2007 and 2008 papers asked unimportant questions, but that the models do not justify the specific conclusions later drawn from them. The FE model was not verified in sufficient detail and was not validated for brain motion or venous response. The implemented loads were not shown to reproduce the head kinematics invoked to justify them. The outputs used to support BV rupture and SDH protection claims also depended on highly simplified BV and BESS assumptions. Together, these problems clarify why the inference is weak.

The loading critique is therefore stronger than a simple mismatch in severity. The shaking input came from a surrogate whose resistance-free sagittal hinge neck was designed as a worst-case upper bound, whereas the comparison impact omitted rotation and represented a much lower-severity event than the surrogate impact it purported to stand beside. The comparison is therefore uneven: one loading condition tends to amplify inertial motion, while the other is reduced. Similarity in spring strain under those circumstances cannot be generalized to real shaking versus real impact.

The bridging-vein critique is likewise broader than rate dependence alone. Representing these vessels as linear springs ignores geometry, orientation, cuff mechanics, and rate sensitivity, yet the inference that shaking alone can rupture them hinges entirely on spring strain. Macroscopic and histological examinations report a free “bridging” segment with minimal supportive meningeal tissue in neonates and infants, thinner walls in early life (as thin as 5–7 µm), and relatively more supportive tissue around adult bridging veins (Cheshire et al., 2024). Such anatomy is not represented by taut, linear springs and likely alters load transfer, slack-to-taut transitions, and local stress concentrations near the sinus cuff.

A more realistic constitutive description is required before the numerical values can be interpreted as evidence of tearing.

The BESS study has a different weakness. Uniformly enlarging the CSF layer to extreme values while leaving tissue properties unchanged already misrepresents clinical heterogeneity, but the decisive issue is the zero pre-strain assumption. Once the bridging veins are allowed even modest baseline strain or reduced slack, the reported damping effect reverses. The protective conclusion for BESS therefore depends on a single favourable initialization assumption rather than on a robust model trend.

### 4.1 A hierarchy of model credibility

Subsequent pediatric FE efforts have emphasized verification and validation against targeted experiments. For impacts (Roth et al., 2010), reported global validation of skull deformability and fracture reproduction. For inertial loading, multibody and FE frameworks have identified neck stiffness and kinematic coupling as dominant drivers of head motion (Wolfson et al., 2005; Jones et al., 2015). Three separate questions should be answered: was the numerical model verified through mesh and parameter checks, were key internal outputs validated against experimental data or credible surrogates, and did the implemented loading actually reproduce the global head kinematics of the event it was claimed to represent? The 2007–2008 papers do not adequately answer any of these questions for the responses on which their central claims depend.

### 4.2 Why the BESS conclusion is unstable

The interpretation that larger CSF spaces reduce BV strain rests on a zero pre-strain assumption and uniform CSF widening. Geometric analysis and clinical context suggest the opposite: increased extra-axial space increases the lever arm for skull–brain relative motion and predisposes to earlier tautening of veins near the superior sagittal sinus for a given brain translation (Papasian and Frim, 2000). Because the model result reverses under plausible alternative initialization, and because the preferred initialization conflicts with anatomy and clinical observation, the protective conclusion of (Raul et al., 2008) is not robust.

### 4.3 What later kinematic work changes

Fixed-ICOR implementations simplify head motion but neglect measured spatiotemporal variations (Schiks et al., 2023). Because BV strain depends on relative skull–brain kinematics and the locus of rotation, fixed-ICOR inputs can bias both the magnitude and distribution of predicted deformation. Incorporating measured kinematics with a moving ICOR is likely to alter BV strain patterns and is best regarded as a minimum realism requirement for shaking simulations. In addition, neck stiffness and grasp configuration couple translation and rotation; peak kinematics in short falls and many activities often coincide with contact events rather than free-air motion (Bertocci et al., 2003; Coats and Margulies, 2008; Ibrahim and Margulies, 2010). Later experiments therefore describe a different physical situation from the one represented by the 2007 input design.

### 4.4 Generalizability beyond a single specimen

Infant heads vary in suture/fontanelle morphology and stiffness, which modulate global compliance and local strain fields (Chatelin et al., 2012; Chen, 2025). Single-anatomy models with fixed material properties are therefore insufficient for generalization. Even if one accepted the numerical output of the original simulations at face value, a single anatomy with a single parameter set could not justify broad population-level claims about infant vulnerability or protection. Sensitivity and uncertainty analyses over plausible anatomical and material ranges are needed to avoid overconfident conclusions.

### 4.5 Limitations

Our reassessment relies on the published descriptions of the original models and first-order recalculations using reported inputs. We did not rerun the original models, and some parameter ranges remain uncertain. Literature integration was targeted rather than systematic. These constraints may understate or overstate specific sensitivities; however, the central critique does not depend on fine parameter tuning. The main conclusions are not adequately supported even before any precise threshold dispute is required.

### 4.6 Requirements for future modelling

To improve the evidentiary basis for infant shaking biomechanics, priority should be given to acquiring infant-relevant bridging-vein constitutive data, including geometry, slack, cuff mechanics, and viscoelasticity under cyclic loading, so that mechanism-appropriate failure metrics can be developed (Famaey et al., 2015). High-fidelity head–neck kinematics of shaking should be measured and published, including moving ICOR, out-of-plane motion, and contact events, so that realistic inputs and validation corridors are available (Schiks et al., 2023). Paediatric FE VandV benchmarks for inertial loading are needed, such as skull–brain relative motion and membrane tensions, analogous to existing impact validation (Roth et al., 2010). Quantifying BESS and extra-axial variability together with vein pre-strain *in vivo* from imaging-derived geometry would help assess risk amplification versus damping (Papasian and Frim, 2000). Studies should also propagate uncertainty across anatomy, materials, and loading rather than presenting single-run determinations as dispositive.

Model-based risk conclusions should be confronted with clinical epidemiology and imaging evidence; for BESS, such clinical data support an increased risk of subdural collections and thus weigh against the specific conclusion of (Raul et al., 2008).

## 5 Conclusion

The two 2007–2008 finite-element papers do not provide sufficiently credible modelling evidence for the conclusions later attributed to them. The model was not verified in detail, was not validated for brain motion or venous response, and was not shown to reproduce the global head kinematics of the surrogate experiments used to motivate its loading. The shaking input inherited a worst-case hinge-neck surrogate, the impact comparator was lower-severity and non-rotational, bridging veins were reduced to linear springs lacking anatomical directionality and rate sensitivity, and the BESS result depended on a biologically insecure zero pre-strain assumption.

Accordingly, the evidence presented in these two papers is insufficient to confirm that non-impact shaking produces impact-level venous loading, or that benign enlargement of the subarachnoid space reduces that loading. Clinical literature, moreover, consistently indicates increased prevalence of subdural collections in infants with BESS (Kapila et al., 1982; Zahl et al., 2011; 2011; Greiner et al., 2013; Tucker et al., 2016; Caré, 2021; Alshareef et al., 2022), contradicting the protective interpretation of (Raul et al., 2008).

Sufficient modelling evidence would require matched-severity inputs across scenarios. It would also require measured head–neck kinematics with moving centres of rotation and contact events where relevant, plus a venous representation that includes slack, cuff geometry, and rate dependence. Validation of skull–brain relative motion and venous surrogates, together with explicit uncertainty quantification over anatomy, materials, and loading, would also be necessary. Until such criteria are met, outputs from similarly simplified models should not be used as stand-alone evidence to assert bridging-vein rupture from non-impact shaking or to claim a protective effect of increased subarachnoid space in BESS.

## Data Availability

The original contributions presented in the study are included in the article/supplementary material, further inquiries can be directed to the corresponding author.
